# Comparative performance of single-use flexible ureteroscopes with and without direct in-scope suction: a retrospective analysis

**DOI:** 10.25122/jml-2026-0042

**Published:** 2026-07

**Authors:** Petrisor Geavlete, Razvan Multescu, Cristian Mares, Cosmin Ene, Catalin Bulai, Bogdan Geavlete

**Affiliations:** 1 Carol Davila University of Medicine and Pharmacy, Bucharest, Romania; 2 Department of Urology, Sf. Ioan Clinical Hospital of Emergency, Bucharest, Romania

**Keywords:** single-use flexible ureteroscopes, direct in scope suction, working channel

## Abstract

Ureteroscopy represents the primary therapeutic approach for a broad spectrum of upper urinary tract stones. The aim of this study was to compare the efficiency of various models of single-use flexible ureteroscopes, some of them with direct in-scope suction (DISS) capabilities. A retrospective study was conducted over a 16-month period, including 75 consecutive patients who underwent retrograde intrarenal surgery for renal lithiasis. Patients were allocated into three groups according to the flexible ureteroscope used: a 7.5 Fr single-use ureteroscope without suction (Group A), a 7.5 Fr single-use ureteroscope with direct in-scope suction (Group B), and a 9.2 Fr single-use ureteroscope with a 5.1 Fr working channel and direct in-scope suction (Group C). Perioperative outcomes, residual stone fragments, and subjective intrarenal visualization were evaluated and compared among groups. A total of 75 patients were included, with stones most frequently located in the renal pelvis (40.0%), followed by the inferior (30.6%), middle (21.3%), and superior calyces (8.0%). Residual stone fragments were observed in 24.0% of cases in Group A, 16.0% in Group B, and 4.0% in Group C; although these differences did not reach statistical significance (*P* = 0.132), a numerical trend toward improved stone clearance was noted with increasing ureteroscope caliber and the use of direct in-scope suction. Subjective intrarenal visualization differed significantly among groups, with the 5.1 Fr working-channel ureteroscope (Group C) achieving significantly higher visualization scores compared with both 7.5 Fr devices (*P* < 0.001). Operative time, hospitalization duration, and postoperative complication rates were comparable across groups. In this retrospective comparative analysis, single-use suction-enabled flexible ureteroscopes demonstrated safety and operative efficiency comparable to those of conventional single-use devices. Although differences in residual fragment rates did not reach statistical significance, improved stone clearance seems to correlate with increasing ureteroscope caliber and the integration of DISS. The 9.2 Fr ureteroscope with a 5.1 Fr working channel provided significantly superior intrarenal visualization, potentially facilitating more efficient lithotripsy.

## Introduction

Ureteroscopy is the primary therapeutic approach for a broad spectrum of upper urinary tract stones; however, conventional techniques have limited ability to achieve complete stone clearance [1]. The extraction of stone fragments using retrieval baskets can be laborious, extending operative time without a significant improvement in stone-free rates (SFRs) [2], whereas lasers are efficient but compromise visibility during the lithotripsy procedure [3]. In this context, despite advancements in laser technology, residual fragments (RFs) and suboptimal intra-operative visibility persist as notable challenges in flexible ureteroscopy [4].

The direct in-scope suction (DISS) ureteroscopy technique has emerged as an innovative solution to overcome these limitations by incorporating an active suction mechanism directly within the ureteroscope [5]. These devices, initially with a 3.6 Fr working channel, demonstrated good visibility, maneuverability, and suction quality, with excellent operative results [6,7]. Fully integrated DISS ureteroscopes constitute a notable advancement in endourological instrumentation. These single-use digital devices are specifically designed with an inherent suction system, eliminating the need for external attachments and thereby optimizing procedural efficiency [7-12].

The quite recent 5.1 Fr working channel DISS ureteroscope allows stone particles up to 250 μm to be suction-evacuated without blockage, even with laser fiber occupancy. A smaller 150 μm fiber size enables higher particle evacuation rates. Urologists should therefore aim for a dust particle size of ≤ 250 μm during routine DISS with the 5.1 Fr working-channel ureteroscope to achieve effective intraoperative stone evacuation [8].

Higher intrarenal pressures are expected with the 9.2Fr scope compared with the 7.5Fr scope, regardless of irrigation flow and ureteral access sheath size. A rapid and significant reduction in pressure occurs only after 1 second of suction activation. This effect is more pronounced with the 9.2Fr ureteroscope, which has a wider working channel measuring 5.1Fr [13].

Despite these technological advancements, comparative clinical evidence evaluating the real-world performance of single-use ureteroscopes with and without integrated DISS remains limited. Most available studies focus predominantly on technical parameters—such as irrigation dynamics, intrarenal pressure modulation, or particle evacuation capacity—while data assessing whether these advantages translate into clinically relevant outcomes, including residual fragment rates, operative efficiency, and perioperative safety, are relatively scarce.

The Pusen 7.5 Fr Digital Single-Use Flexible Ureteroscope PU3033AH (ZhuHai PusenMedical Technology Co., Ltd., Guangdong, China) has the add-on suction accessory directly attached to the scope through the working channel. This allows clinicians to activate the suction to remove dust and small fragments during the procedure [14].

The Pusen 5.1 Fr working channel model with a 6.8 Fr distal tip (PU400) has been designed to facilitate improved visualization through active field clearance using DISS, enabling rapid deployment of suction while potentially reducing intrarenal pressure and temperature during lithotripsy.

In this context, the primary objective of our study was to conduct a real-world comparative evaluation of intraoperative performance, visualization quality, and perioperative outcomes among three single-use flexible ureteroscopes, including two devices with DISS capabilities.

Based on previously published experimental and early clinical observations suggesting that suction-enabled ureteroscopes and larger working channels may improve intrarenal visualization and facilitate fragment evacuation, the present study aimed to explore whether these technical advantages translate into measurable clinical benefits, including residual fragment rates, operative efficiency, and perioperative safety, in routine endourological practice.

A secondary objective was to explore whether differences in device design characteristics, such as working channel diameter and suction integration, may influence clinical outcomes in routine endourological practice.

## Material and Methods

A 16-month retrospective study was carried out between May 2024 and September 2025, including 75 consecutive eligible patients treated for renal lithiasis. The study’s inclusion criteria were patients aged 18 or older with solitary intrarenal stones up to 25 mm in size that were not staghorn stones, without upper urinary system abnormalities, and who had not undergone prior urological interventions for lithiasis.

Patient allocation to each study group was not randomized and was determined primarily by device availability during the study period, reflecting real-world adoption of very new suctioning devices in clinical practice. As such, the study design is subject to potential selection bias because treatment assignment was not based on predefined clinical criteria. However, to minimize this risk, all patients were included consecutively according to predefined inclusion and exclusion criteria, and all procedures were performed by the same highly experienced surgical team using standardized operative protocols.

Written informed consent, including consent for anonymized scientific use of clinical data, was obtained from all patients at the time of treatment. The present study represents a retrospective analysis of these clinical data. The study protocol for retrospective data review was approved by the hospital’s Ethics Committee (approval no. 15977/15.09.2025) and was conducted in accordance with the Declaration of Helsinki.

### Collected data

Demographic information, including sex, age, and medical and surgical history, was obtained from the medical files. Additionally, information on the location, dimensions, and volume of the stones, along with their density in Hounsfield units (HU), was recorded. During the operation, the visualization quality was evaluated subjectively by the experienced urologist who conducted all the procedures. Postoperative morbidity, along with the duration of hospitalization, was also assessed. In all cases, kidney-ureter-bladder radiography (KUB) and renal ultrasonography were performed 3 weeks postoperatively to assess stone-free status. Non-enhanced CT was also performed each time when residual fragments >3 mm were identified.

### Statistical analysis

Statistical analyses were performed using IBM SPSS Statistics for Windows version 26.0 (IBM Corp., Armonk, NY, USA) and Microsoft Excel. Continuous variables were expressed as means with standard deviations, while categorical variables were presented as frequencies and percentages.

Data distribution was assessed using the Shapiro–Wilk test to determine normality. For comparisons between the three groups, normally distributed continuous variables were analyzed using one-way analysis of variance (ANOVA), while non-normally distributed variables were evaluated using the Kruskal–Wallis test. When statistically significant differences were identified, post-hoc pairwise comparisons were performed.

Categorical variables were compared using the Chi-square test or Fisher’s exact test when appropriate. A *P* value < 0.05 was considered statistically significant.

Given the retrospective design of the study, no a priori sample size calculation was performed. Therefore, the statistical analyses should be interpreted as exploratory, and the results should be considered hypothesis-generating.

### Study design

**Figure 1:**
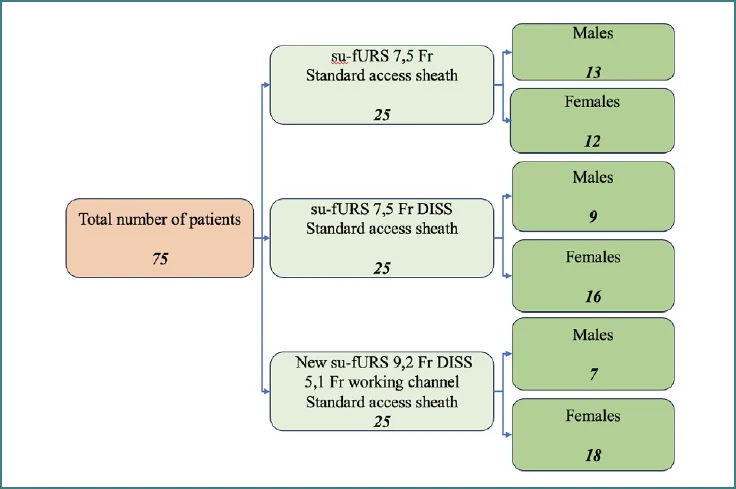
Flowchart of patients selection in the present study

**Figure 2:**
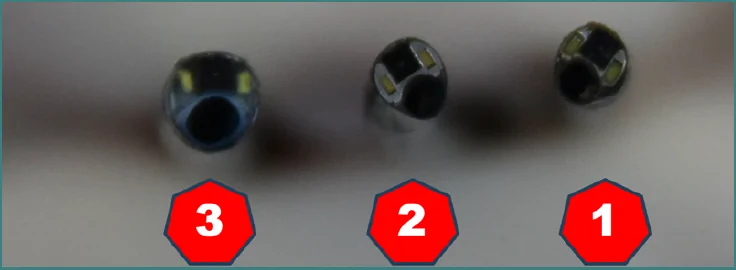
Comparative visual analysis of the su-fURS tips (from right to left: 1. Pusen PU 3033A, 2. Pusen 3033 AH, 3. Pusen PU400 – to be noticed, despite the overall 9,2 Fr width of the PU400 scope, the tip measures only 6,8 Fr).

**Figure 3:**
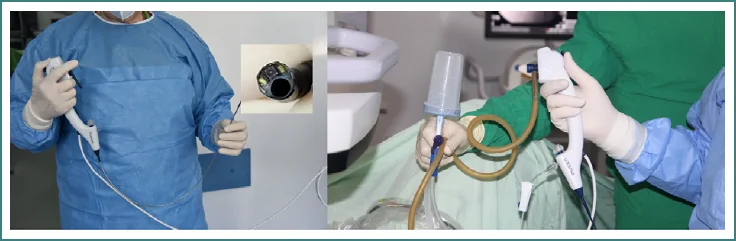
PU400 scope (9,2 Fr with a 5,1 Fr working channel)

A retrospective study was undertaken including 75 consecutive patients who underwent retrograde intrarenal surgery (RIRS) using different technical approaches, as detailed below: Group A included 25 patients who underwent RIRS with a single-use 7.5 Fr flexible ureteroscope without aspiration, using a conventional 10/12 Fr ureteral access sheath; Group B included 25 patients who underwent RIRS with a single-use 7.5 Fr flexible ureteroscope with DISS, also using a conventional 10/12 Fr ureteral access sheath; Group C included 25 patients who underwent RIRS with a new 9.2 Fr ureteroscope with DISS, featuring a 5.1 Fr working channel, also used in conjunction with a conventional 10/12 Fr ureteral access sheath (Figures 1–3).

### Technique and devices

Operative technique, irrigation strategy, laser settings, and access sheath usage were standardized across all study groups to minimize procedural variability.

**Figure 4:**
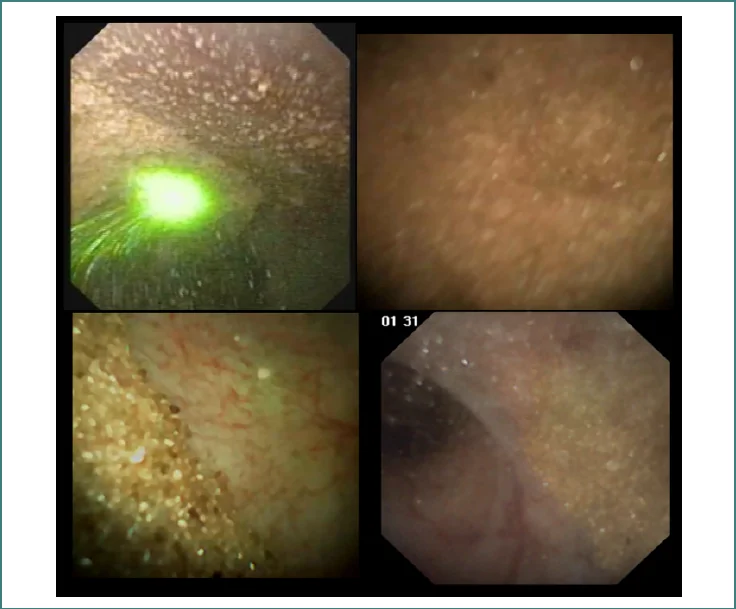
Intraoperative visualization using Pusen PU 3033AH in patients from Group B

**Figure 5:**
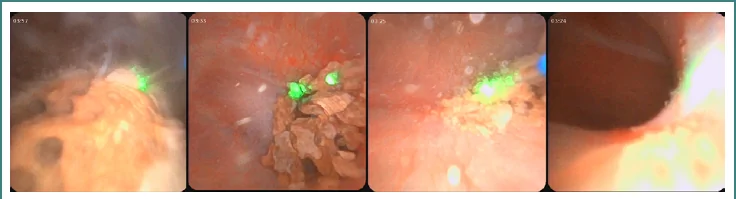
Intraoperative visualization using Pusen PU 400 in patients from Group C

In all cases, spinal anesthesia was performed, and the patient was placed in the lithotomy position. Urethrocystoscopy was performed at the beginning of each procedure to catheterize the ureteral orifices with a stiff hydrophilic guidewire with a diameter of 0.035 inches, which is inserted into the renal cavities under fluoroscopic guidance. In all cases, we initially performed semirigid ureteroscopy to thoroughly inspect the ureter and exclude lesions at this level, thereby allowing proper insertion of the ureteral access sheath. The use of a 10/12 Fr ureteral access sheath and operative steps were standardized across all cases. Subsequently, under direct visualization, we advanced the flexible ureteroscope onto the sheath and performed a pyelocalicoscopy to fully evaluate the pyelocaliceal system, identify the calculus described on preoperative imaging, and evaluate any other lesions, if present, within the collecting system. Following stone identification, the Richard Wolf MegaPulse 70+ Holmium: YAG laser was used for stone lithotripsy in all cases, together with a single-use 250-mm laser fiber. We predominantly used renal dusting settings, with a pulse energy of 0.5 J and a frequency of 40–60 Hz. This configuration was routinely applied during intrarenal lithotripsy to achieve efficient stone pulverization with minimal retropulsion. In all cases, at the end of each surgery, all renal calyces were inspected to exclude significant residual lithiasis (Figures 4 and 5), and a double J stent was inserted. The single surgeon who performed all surgical interventions subjectively evaluated the visualization of the pyelocaliceal environment in terms of operative field clarity using a 5-point Likert-type scale (0 = poorest, 4 = optimal).

## Results

A total of 75 consecutive patients were evaluated, depending on instrument availability, in three distinct groups of 25 patients each, according to the described protocol, comprising 46 females (61.33%) and 29 males (38.66%). The men ranged in age from 22 to 72 years, with a mean of 48.4 years, and the women ranged from 25 to 69 years, with a mean of 45.9 years. Overall, the most frequent stone location was the renal pelvis (30 cases), representing 40.0% of the total number, followed by the inferior calyx (23 cases), representing 30.6%, the middle calyx (16 cases), representing 21.33%, while the fewest cases were in the superior calyx (6 cases), representing 8.0%. Table 1 displays the stone locations for all assessed patients.

**Table 1:** Assessment of the location of kidney stones in each investigated group

Stone position	Group A		Group B		Group C		Total	
	*n*	%	*n*	%	*n*	%	*n*	%
Renal pelvis	10	40.0	9	36.0	11	44.0	30	40.0
Inferior calix	7	28.0	7	28.0	9	36.0	23	30.6
Middle calix	7	28.0	6	24.0	3	12.0	16	21.33
Superior calix	1	4.0	3	12.0	2	8.0	6	8.0

Regarding stone characteristics, the following parameters were assessed: length, width, height, volume (mm^3^), and stone density measured on computed tomography (CT) in Hounsfield units (HU). In Group A, stone length ranged from 10 to 25 mm, with a mean of 18.56 mm. Stone volume ranged from 125 to 1810 mm^3^, with a mean of 710.82 mm^3^. Stone density ranged from 440 to 1375 HU, with a mean of 959.8 HU. In Group B, stone length ranged from 14 to 25 mm, with a mean of 18.6 mm. Stone volume ranged from 190 to 1482 mm^3^, with a mean of 658.22 mm^3^. Stone density ranged from 440 to 1410 HU, with a mean of 1001.6 HU. In Group C, stone length ranged from 12 to 25 mm, with a mean of 19.6 mm. Stone volume ranged from 180 to 1596 mm^3^, with a mean of 793.86 mm^3^. Stone density ranged from 450 to 1400 HU, with a mean of 1000.8 HU. Across the entire study cohort, stone length ranged from 10 to 25 mm, with a mean of 19.04 mm. Stone volume ranged from 125 to 1810 mm^3^, with a mean of 720.96 mm^3^. Stone density ranged from 440 to 1410 HU, with a mean of 987.4 HU. A complete overview of the stone characteristics of all patients included in the study is presented in Table 2.

**Table 2:** Assessment of dimensions, volumes, and stone density in the analyzed groups

	Group A			Group B			Group C			Total		
	Min	Max	Average	Min	Max	Average	Min	Max	Average	Min	Max	Average
Length (mm)	10	25	18.56	14	25	18.6	12	25	19.96	10	25	19.04
Width (mm)	4	14	9.36	5	13	9.56	5	14	9.72	4	14	9.54
Height (mm)	3	12	7.36	3	13	7.04	4	12	7.84	3	13	7.41
Volume (mm^3^)	125	1810	710.82	190	1482	658.22	180	1596	793.86	125	1810	720.96
Stone density (HU)	440	1375	959.8	440	1410	1001.6	450	1400	1000.8	440	1410	987.4

Comparative analysis of baseline characteristics, including stone size, volume, density, and location, demonstrated no statistically significant differences between the three groups (all *P* > 0.05), confirming baseline comparability.

The primary objective of this study was to compare the intraoperative performance, visualization quality, and perioperative outcomes of three different single-use flexible ureteroscopes used during intrarenal lithotripsy.

Regarding the total operating time (from cystoscopy to Foley catheter insertion), in Group A, the minimum was 30 minutes, the maximum was 90 minutes, and the average was 53.2 minutes. In Group B, the minimum operating time was 35 minutes, the maximum was 80 minutes, and the average was 55.0 minutes. In Group C, the minimum operating time was 30 minutes, the maximum was 75 minutes, and the average was 53.56 minutes.

Another important aspect to evaluate in the present study was the average hospitalization time: 1.24 days in Group A, 1.16 days in Group B, and 1.08 days in Group C.

One of the main pillars in the evaluation of any surgical instrument is the report of postoperative complications. In the current evaluation, in Group A, four patients (16.0%) presented hematuria (Clavien I), and three patients (12.0%) presented fever (Clavien II) in the first 24 hours postoperatively. In Group B, three patients (12.0%) presented with hematuria (Clavien I), while only one patient (4.0%) presented with fever (Clavien II) on the first postoperative day. In Group C, only one patient presented with hematuria (Clavien I) and only one with fever (Clavien II) (4.0%) (*P* = 0.411).

Stone-free status was assessed in all patients three weeks after the procedure using renal ultrasonography and kidney–ureter–bladder (KUB) radiography, with additional non-contrast computed tomography (CT) performed in selected cases. Residual stone fragments larger than 3 mm were considered clinically significant. When such fragments were identified, non-contrast CT was performed to further evaluate them and guide subsequent management. Residual stones after lithotripsy were identified in six patients (24.0%) in Group A, four patients (16.0%) in Group B, and one patient (4.0%) in Group C (*P* = 0.132).

Another important aspect of this comparative evaluation of the three single-use flexible ureteroscopes was the assessment of image quality through direct intraoperative visualization. The Pusen 3033A ureteroscope used in Group A achieved a mean visualization score of 3.04, similar to that of the Pusen 3033 AH ureteroscope used in Group B (3.20). In contrast, the Pusen PU400 ureteroscope used in Group C, with its larger outer diameter and substantially wider working channel, provided superior visualization, achieving a mean score of 3.72. Subjective intrarenal visualization differed significantly among the three groups (Kruskal–Wallis H = 48.7, *P* < 0.001) (Figures 4 and 5). Post hoc analysis demonstrated no significant difference between Groups A and B (p = 0.88), whereas Group C showed significantly higher visualization scores than both Groups A and B (*P* < 0.001). A complete comparison of the outcomes across the three groups is presented in Table 3.

**Table 3:** Assessment of the outcomes based on operative duration, complications, length of hospital stay, use of postoperative stents, residual stone presence, and subjective evaluation of visualization during lithotripsy

	Group A			Group B			Group C		
Complications	Hematuria – four cases Fever – three cases			Hematuria – three cases Fever – one case			Hematuria – one case Fever – one case		
Operative time (min)	Min	Max	Average	Min	Max	Average	Min	Max	Average
	30	90	53.2	35	80	55.0	30	75	53.56
Postoperative ureteral stent Number of cases (percentage)	9 (36.0)			6 (24.0)			4 (16.0)		
Hospital time (days – average)	1.24			1.16			1.08		
Residual stones Number of cases (percentage)	6 (24.0)			4 (16.0)			1 (4.0)		
Subjective analysis of visualization	Mean 3.04			Mean 3.20			Mean 3.72		

Visualization was assessed using a subjective 5-point scale (0–4), with higher scores indicating better visualization during lithotripsy

## Discussion

Our study included 75 consecutive patients undergoing flexible ureteroscopy, with a mean age of 46.8 years and a predominance of females (61.33%). The demographic distribution was comparable to that reported in epidemiological studies of renal stone disease, in which middle-aged adults are the most affected group [15]. Renal pelvis stones were the most frequent (44%), followed by inferior calyceal (26.66%) and middle calyceal stones (21.33%), a pattern consistent with previous reports indicating that the renal pelvis and lower pole are the most common sites targeted during flexible ureteroscopic intervention [16]. In our study, stone dimensions and density were heterogeneous, with mean stone lengths ranging from 18.56 mm to 19.6 mm and mean densities approaching 1,000 HU, values comparable with existing literature where typical stone densities in fURS-treated calculi range between 900 and 1,100 HU [17,18] in similar study models.

As stated before, all procedures were performed by the same experienced endourological surgical team with extensive expertise in flexible ureteroscopy, following a standardized operative protocol. This approach aims to minimize variability in surgical technique, laser settings, and intraoperative decision-making, thereby reducing operator-dependent confounding factors.

Residual fragment rates differed numerically among the three groups (24% in Group A, 16% in Group B, and 4% in Group C), indicating a trend toward improved stone clearance with increasing ureteroscope caliber and the use of direct in-scope suction. However, these differences did not reach statistical significance, likely due to the limited sample size. Our findings align with evidence indicating that suction-assisted flexible ureteroscopy may enhance fragment evacuation and improve stone-free rates, as reported by Patel *et al*., who observed superior outcomes with in-scope suction systems compared with conventional techniques [5].

Stone location is a well-recognized factor influencing the success of flexible ureteroscopy and the likelihood of residual fragment persistence. In the present study, stones were most frequently located in the renal pelvis and lower calyx, anatomical sites that present different technical challenges during endoscopic management. Although a formal subgroup analysis was not performed due to the limited sample size, it is plausible that the higher proportion of lower calyceal stones (characterized by unfavorable anatomical angles and more difficult access) may have contributed to the occurrence of residual fragments in some cases. This anatomical factor should be considered when interpreting comparative stone-free outcomes, as it may influence fragment clearance independently of ureteroscope design or suction capability.

Our operative times were similar across all three groups, averaging 53.2 minutes in Group A, 55.0 minutes in Group B, and 53.56 minutes in Group C, indicating that the use of advanced instrumentation did not significantly prolong surgical duration, a finding consistent with systematic reviews showing minimal differences in operative time when suction systems are employed [19].

In our study, hospitalization duration remained short across all groups, ranging from 1.08 to 1.24 days, reflecting the minimally invasive nature and rapid postoperative recovery associated with flexible ureteroscopy, as also reported by Huang *et al*. in large retrospective cohorts [20].

Regarding postoperative complications, we observed hematuria in 16% of patients in Group A, 12% in Group B, and 4% in Group C, and postoperative fever in 12%, 4%, and 4% of patients, respectively. This progressive reduction in complication rates suggests that improved irrigation dynamics and enhanced fragment evacuation reduce intrarenal pressure and, consequently, infectious risk. Previous studies have established that increased intrarenal pressure and retained stone fragments are key contributors to postoperative fever and infectious complications, and that suction-assisted systems help reduce these risks by optimizing renal pelvic pressure control [21].

In our study, visualization quality was subjectively assessed by the operating surgeon. The enhanced visualization may contribute to improved stone-free rate by facilitating more precise targeting and efficient fragmentation of calculi. Similar findings have been reported in technical assessments of modern flexible ureteroscopes, in which larger working channels and improved irrigation systems were shown to significantly enhance optical performance and procedural precision [22].

Adequate intrarenal visualization is a critical determinant of procedural efficiency, safety, and clinical outcomes in ureteroscopic interventions. In the present analysis, visualization quality was assessed using a surgeon-reported subjective scale that reflects real-time operative conditions. Although subjective, such an assessment reflects real-world intraoperative conditions; however, objective quantitative measures would strengthen future analyses. The findings demonstrate that both the Pusen 3033A (Group A) and Pusen 3033 AH (Group B) ureteroscopes provided comparably high visualization quality, with mean scores of 3.04 and 3.20, respectively. The absence of a statistically significant difference between these two instruments suggests that modest variations in design, such as irrigation and aspiration capabilities, may not substantially affect perceived image quality when the caliber and working channel dimensions are similar. This observation aligns with previous reports indicating that image resolution and optical system quality often outweigh minor differences in fluid dynamics when scope diameter is comparable [23]. In contrast, the Pusen PU 400 ureteroscope (Group C) achieved significantly higher visualization scores than both Groups A and B. This improvement is likely attributable to the wider caliber and substantially enlarged working channel, which enhance irrigation flow, reduce intrarenal debris accumulation, and maintain clearer operative fields. Prior studies have shown that increased irrigation efficiency is directly associated with improved visualization, reduced operative time, and higher stone-free rates during flexible ureteroscopy [24]. The statistically significant difference observed among the three groups of our study underscores the impact of instrument design on visualization quality. Importantly, while Groups A and B demonstrated “very good” visualization, the markedly higher scores in Group C suggest that larger caliber ureteroscopes may offer clinically relevant advantages, particularly in complex cases involving stone burden, bleeding, or prolonged operative times [25]. However, these benefits must be weighed against potential drawbacks, such as increased risk of ureteral trauma or the need for ureteral access sheath placement, which were not addressed in the present evaluation. A key limitation of this assessment is its reliance on subjective surgeon scoring, which may introduce observer bias. Nonetheless, subjective visualization scales have been validated in previous ureteroscopic research and remain valuable when objective optical metrics are unavailable. Future studies incorporating objective measures—such as digital image analysis, flow rate quantification, or multi-surgeon assessments—would strengthen the evidence base and improve generalizability [26]. In summary, while all evaluated ureteroscopes provided clinically acceptable visualization, the Pusen PU 400 demonstrated a statistically and clinically significant advantage. These findings highlight the importance of scope caliber and working channel size in optimizing intrarenal visualization and may inform instrument selection in endourological practice. From a clinical perspective, suction-enabled ureteroscopes may be particularly beneficial in cases with higher stone burden, limited visibility, or when efficient fragment evacuation is critical. However, these potential advantages must be weighed against the higher cost of single-use devices, which remains an important consideration in real-world practice and healthcare resource allocation.

Several methodological limitations of this study must also be acknowledged. First, the retrospective and non-randomized design inherently introduces potential selection and information biases and may limit the ability to clearly establish causal relationships between device characteristics and clinical outcomes. Although consecutive patient inclusion and standardized surgical protocols were applied to mitigate this bias, residual confounding cannot be excluded. Patient allocation to each study group was determined primarily by device availability during the study period. This allocation pattern reflected the progressive introduction of newer suction-enabled ureteroscopic technologies into routine clinical practice rather than predefined selection criteria or surgeon preference. While this reflects real-world technology adoption, such allocation may still introduce potential selection bias that should be considered when interpreting comparative outcomes. A potential temporal bias, including a learning-curve effect, cannot be entirely excluded, although all procedures were performed by an experienced surgical team using standardized techniques over a 16-month timeframe, making this improbable.

Second, the relatively modest sample size may have limited the statistical power to detect small but clinically meaningful differences between groups, particularly regarding residual fragment rates and postoperative complications. Consequently, the absence of statistically significant differences should be interpreted with caution, as the study was primarily exploratory and hypothesis-generating.

Third, postoperative assessment of stone-free status was based predominantly on ultrasonography and kidney-ureter-bladder radiography, with non-contrast CT performed selectively. While this approach reflects routine clinical practice, it may underestimate residual fragments, particularly those ≤3 mm. Although the likelihood of such small fragments becoming clinically significant is very low, this does not rule out the risk of classification errors in evaluating stone-free rates.

Additionally, intrarenal visualization quality was assessed subjectively by the operating surgeon using a Likert-type scale, potentially introducing observer bias. Although such subjective evaluation reflects real-world operative conditions, objective quantitative measures of visualization were not available.

Although multivariate analysis could provide additional adjustment for potential confounders, the relatively small sample size and limited number of outcome events may lead to model overfitting and unreliable estimates. Therefore, no multivariate model was performed, and the findings should be interpreted as exploratory.

Finally, all procedures were performed by a highly experienced surgical team using standardized techniques, which, although it reduces operator variability, may limit the generalizability of the findings to centers with different levels of expertise or procedural protocols.

## Conclusion

In this retrospective comparative analysis, single-use suction-enabled flexible ureteroscopes demonstrated safety and operative efficiency comparable to those of conventional single-use devices. Although differences in residual fragment rates did not reach statistical significance, a numerical trend toward improved stone clearance was observed with increasing ureteroscope caliber and the integration of direct in-scope suction. The 9.2 Fr ureteroscope with a 5.1 Fr working channel provided significantly superior intrarenal visualization, which may offer procedural advantages requiring confirmation in prospective studies.

These findings should be interpreted as exploratory and hypothesis-generating, providing real-world insights into the potential advantages of suction-enabled ureteroscopic technology. Further adequately powered, randomized, prospective studies incorporating standardized postoperative imaging and objective intraoperative measurements are required to confirm these observations.
